# The Effect of Electrolytic Jet Orientation on Machining Characteristics in Jet Electrochemical Machining

**DOI:** 10.3390/mi10060404

**Published:** 2019-06-17

**Authors:** Xinmin Zhang, Xudong Song, Pingmei Ming, Xinchao Li, Yongbin Zeng, Jintao Cai

**Affiliations:** 1 Institute of Non-Traditional Machining & Equipment, Henan Polytechnic University, Jiaozuo 454000, China; zhangxm@hpu.edu.cn (X.Z.); hpulixinchao@163.com (X.L.); 15839113078@126.com (J.C.); 2 Jiangsu Key Laboratory of Precision and Micro-Manufacturing Technology, College of Mechanical and Electrical Engineering, Nanjing University of Aeronautics and Astronautics, Nanjing 210016, China; binyz@nuaa.edu.cn

**Keywords:** jet electrochemical machining, electrolytic jet machining, machining localization, horizontal jet orientation, machining accuracy

## Abstract

Jet electrochemical machining (Jet-ECM) is a significant prospective electrochemical machining process for the fabrication of micro-sized features. Traditionally and normally, the Jet-ECM process is carried out with its electrolytic jet being vertically impinged downstream against the workpiece. Therefore, other jet orientations, including a vertically upstream orientation and a horizontal orientation, have rarely been adopted. In this study, three jet orientations were applied to electrolytic jet machining, and the effect of jet orientations on machining characteristics was systemically investigated. Horizontal jet orientation is of great benefit in achieving accurate micro-sized features with excellent surface quality with either a static jet or a scanning jet for the Jet-ECM. On the other hand, the Jet-ECM with a horizontal jet orientation has a smaller material removal rate (MMR) than the ones with vertical jet orientations, which have almost the same MMR. It was found that an enhancement of machining localization and a reduction of MMR for horizontal jet electrochemical machining primarily results from an improvement of the mass-transfer field. The horizontal orientation of the jet is beneficial for the Jet-ECM processes to improve machining accuracy.

## 1. Introduction

Jet electrochemical machining (Jet-ECM) is a special form of electrochemical machining (ECM) using an electrolytic jet as a tool. It has some advantages over other electrochemical machining processes. These advantages include good selectivity (localization), a high processing speed, and great flexibility in its machining position. By programming a moving path for the electrolytic jet and controlling process parameters, Jet-ECM can produce a wide variety of micro-scale geometric features such as dimples [1], holes [2], grooves [3], three-dimensional complex structures [4], and surface textures [5]. Additionally, it can accomplish the same tasks as various other machining tools, such as polishing [6], turning [7], milling [8], and cutting [9].

Although Jet-ECM processes have been intensively studied, their machining localization (machining accuracy) and surface quality are still less desirable for precision and ultraprecision machining applications. Thus, up to now, various efforts have been being made to improve the machining localization of Jet-ECM processes. Sen et al. [10] attempted to increase the machining localization of Jet-ECM by raising the feed rate of the jet nozzle and obtained a reduced radial overcut and a smaller taper of the machined holes. It is a common practice to adopt an acidified or acidic solution as the electrolyte to promote the discharge of electrolytic products from the machining area in the Jet-ECM processes to improve machining quality [11,12,13]. However, the use of the acidified or acidic electrolyte usually brings about some problems, such as an increase of equipment development and maintenance costs, a big difficulty in disposing the electrolytic products, and problems renewing the electrolyte, thereby reducing the versatility and applicability of Jet-ECM. Therefore, in recent years, using a neutral electrolyte in Jet-ECM has become more popular [14,15]. J. Mitchell-Smith et al. [16] added NaI to a NaNO_3_ electrolyte to develop an iodide-doped electrolyte for the Jet-ECM process and found that the machining localization is greatly enhanced compared to the NaNO_3_ or NaCl electrolytes. The application of a high frequency ultra-short pulse current and a bipolar pulse current to the electrolyte jet machining was found to facilitate the achievement of mirror-like surfaces on the processed features [6]. M. Datta et al. [17] reported that the coaxial incorporation of a laser beam with the electrolytic jet enabled the reduction of stray-current corrosion. P.T. Pajak et al. [18] further verified that the auxiliary laser beam can greatly increase the material removal rate, and can also improve surface quality and the perpendicularity of the side-wall’s machined micro-scale features. Harsha Goel et al. [19] found that the impinging electrolytic jet, coaxially with the compressed air, is able to produce holes with significantly improved dimensional accuracy. Currently, the addition of coaxial air has been preferably used to enhance the machining accuracy of the Jet-ECM process by some researchers [20]. Very recently, Jonathan Mitchell-Smith et al. [21] studied the effect of the shape of the jet nozzle tip on the current density distribution and machining characteristics during Jet-ECM, and advanced the flexibility and performance of Jet-ECM by varying the angle of the jet address [14].

**Figure 1:**
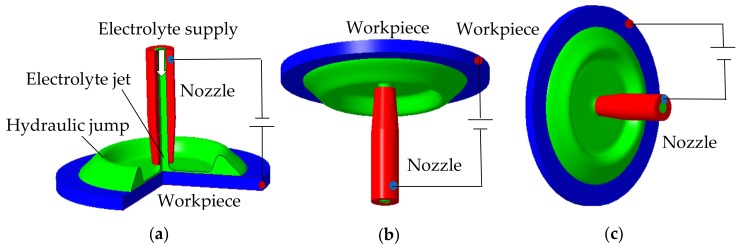
Schematic of jet electrochemical machining with different orientations. (**a**) Vertically downstream orientation; (**b**) vertically upstream orientation; (**c**) horizontal orientation.

Jet-ECM has been presented in various manners for different applications, such as jet electrolytic drilling (JED), electro-stream drilling (ESD), and capillary drilling (CD). Among these processes, JED could be the most popular and widely used Jet-ECM process because of its easy realization of the jet nozzle. The nozzle for the JED process is metallic and commercially available in a variety of shapes and sizes. However, it is a bit troublesome because the outside wall of the nozzle has to be insulated to avoid the electric field connecting with the workpiece. It was shown by Matthias Hackert-Oschätzchen et al. [22] that stray-current corrosion often occurs because of the re-contact of the electrolyte reflected from the workpiece with the jet nozzle. As a result, a variety of efforts have been made by researchers [23] to insulate the outside wall of the metallic nozzle for JED processes. Traditionally, Jet-ECM is carried out in such a way that the nozzle is placed vertically, and the jet is impinged downstream against the workpiece. Here, such an orientation of the jet for traditional Jet-ECM is called the vertically downstream orientation, as shown in Figure 1a. Although this traditional jet orientation is relatively easy to operate, a hydrodynamic jump frequently takes place at the workpiece, especially when the workpiece surface is large and planar, which generally results in an expansion of stray-current corrosion areas. Further, in such a jet orientation, an electrolyte reflection phenomenon is often observed, which, as stated above, is one of the major causes that deteriorates machining localization for serious secondary stray-current corrosion. Accordingly, the reduction, or even elimination, of these two phenomena is very necessary. Since the occurrence of these negative phenomena could be caused by the configuration between the impinging jet and the workpiece, in this study, other configurations were also employed. Further, the machining characteristics of Jet-ECM, carried out with different configurations (shown in Figure 1), were evaluated comparatively. For these aspects, almost no scientific findings have been reported.

## 2. Flow Field and Current Density Distribution Characteristics of Jet-ECM with Different Jet Orientations

In jet-ECM, dissolution behavior and machining characteristics are determined primarily by current density distribution over the machining area, while the current density distribution characteristics are highly dependent on the flow field distribution characteristics of the impinging jet on the workpiece. In order to understand the effect of jet orientation on the flow field and current density distribution during Jet-ECM, a numerical analysis was performed using the commercial software, COMSOL Multiphysics (COMSOL Multiphysics 5.2a, COMSOL Inc., Stockholm, Sweden), and a motion analyzing microscope (VHX-2000, Keyence, Osaka, Japan) was applied to observe and verify the flow field while machining micro-dimples.

### 2.1. Simulation of Flow Field and Current Density Distribution of Electrolyte Jet With Different Orientation

#### 2.1.1. Physical Models

**Figure 2:**
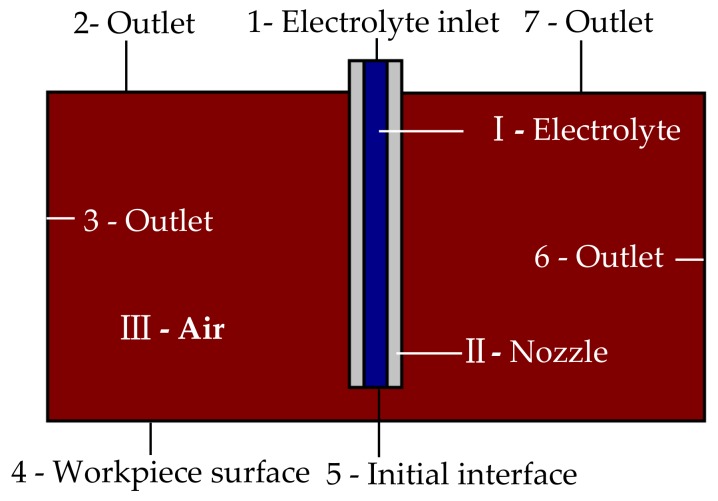
Schematic of the developed 2-D models for the simulations.

Figure 2 shows the schematic of the developed 2-D models for the simulations. Domain I denotes the electrolyte (NaNO_3_ 20wt.%), Domain II denotes the nozzle (material: SUS 304), and Domain III donates the air. Boundary 1 is set as the inlet of the electrolyte, and boundaries 2, 3, 6, and 7 are set as the outlets of the electrolyte. Boundary 4 denotes the workpiece surface (substrate wall boundaries). Boundary 5 is set as the initial interface boundary. Other boundaries are set as wall boundaries.

The Laminar Flow, Two-phase, Level set mode is used to simulate the flow field of Jet-ECM. This procedure was previously implemented by Hackert-Oschätzchen M et al. [21] to study the jet shape in Jet-ECM. The phase characteristic is determined by the level set function Φ. The phase is considered to be the air and electrolyte when Φ = 0 and Φ = 1, respectively; when Φ = 0.5, the phase is considered to be the interface between the air and the electrolyte.

The change of the electrolyte concentration gradient is neglected in Jet-ECM due to the high speed of the electrolyte jet. Therefore, a secondary current distribution physics field is applied to simulate the current density distribution of Jet-ECM.

#### 2.1.2. Governing Equations, Boundary Conditions, and Solutions

The interface moves with the fluid velocity. The convection of the reinitialized level set function is described as follows:

∂Φ/∂t + ∇·(Φu) + γ[(∇·(Φ(1 − Φ)·∇Φ/|∇Φ|)) − ε∇·∇Φ] = 0(1)

where u is the moving velocity of the interface, γ is the initial velocity of the interface, and ε is the thickness of the interface.

In order to define the fluid interface, the level set function is used to smoothen the density and viscosity across the interface by the definitions. The density (ρ) and kinematic viscosity (µ) of the interface are given as follows:

ρ = ρ_air_ + (ρ_elec_ − ρ_air_)Φ (2)

μ = μ_air_ + (μ_elec_ − μ_air_)Φ (3)

where ρ_elec_ and ρ_air_ are the density of the electrolyte and the air, respectively, and μ_elec_ and μ_air_ are the viscosity of the electrolyte and the air, respectively.

The Navier–Stokes equation is used to characterize the mass and momentum transfer of the fluids, including the electrolyte, the air, and their interface. Both the electrolyte and air can be considered incompressible since the fluid velocity is small compared to the speed of sound. The Navier-Stokes equation is expressed as follows:

ρ(∂u/∂t + u·∇u) − ∇·(μ(∇u + ∇u^T)) + ∇p = F_st_ (4)

(∇·u) = 0(5)

where p is pressure (Pa) and F_st_ is surface tension. The surface tension F_st_ can be calculated by the following formula:

F_st_ = ∇·T (6)

T = σ(I − (nn^T))δ(7)

where I is the unit matrix, n is the interface normal, σ is the surface tension coefficient (N/m), δ is the delta function, which is nonzero only at the fluid interface. The estimation formula of the normal interface is presented as follows:

n = ∇Φ/|∇Φ|,(8)

and the delta function is approximately presented in the following manner:

δ = 6|Φ(1 − Φ)|.(9)

Table 1 lists the domain definition and boundary conditions for the numerical simulations. The inlet electrolyte was assumed to be in the laminar state, and its initial velocity was 15 m/s. The outlet pressure was kept at zero.

Table 2 shows material definitions. To ensure numerical stability, the electric conductivity of air is defined as σ_A_ = 1 × 10^−5^ S/m, which is not zero.

Table 3 shows the boundary conditions for the simulations of electrodynamics. The electric potential of the anode (boundary 4) is defined as U_A_ = 20 V, and the electric potential of cathode (nozzle wall) is defined as U_C_ = 0 V. The remaining boundaries are for electric insulation.

The commercial software COMSOL Multiphysics (5.2 version) was used to simulate the flow field and current density distribution of the electrolyte jets with different orientations. To enhance simulation accuracy, the interface was finely meshed in a transient adaptive mode. To better understand the evolution of the flow field and the current density distribution of Jet-ECM while machining micro-dimples, the simulation results on three machining phases (the initial phase (in which the dimple is not drilled), the shallow-dimple phase (in which the aspect ratio of dimple is 0.1), and the deep-dimple phase (in which the aspect ratio of dimple is 0.25)) were examined. The shapes of the dimples in different phases were obtained from the experiment.

#### 2.1.3. Results and Discussion

**Figure 3:**
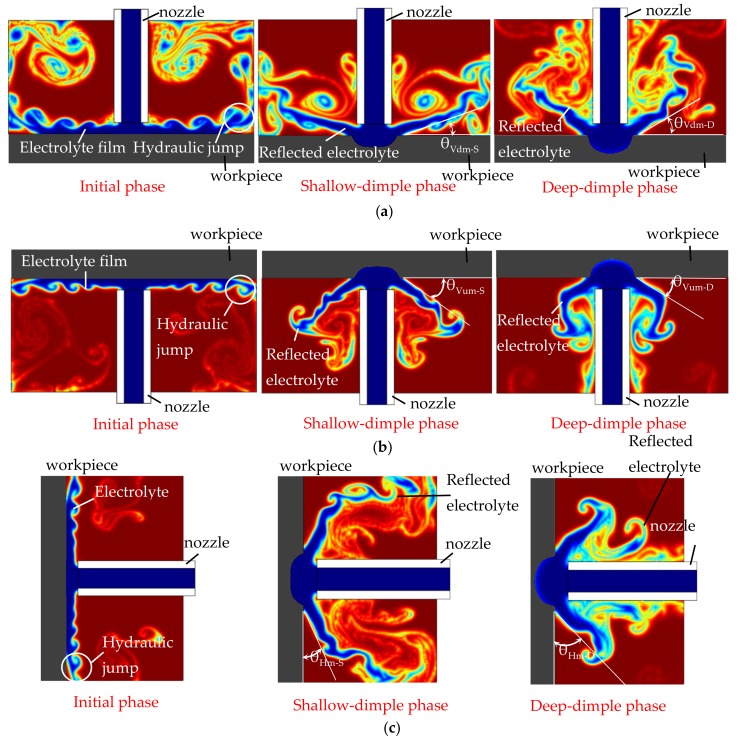
Simulated results of flow field distribution during Jet-ECM in different phases. (**a**) Vertically downstream orientation; (**b**) vertically upstream orientation; (**c**) horizontal orientation.

In order to understand the effect of the jet orientation on the electrolyte flow behaviors and current density distribution during Jet-ECM, a simulation was conducted. Figure 3 shows the simulated results of the flow field during Jet-ECM with three orientations: the vertically downstream orientation, the vertically upstream orientation, and the horizontal orientation. Generally, the reflection of the impinged electrolyte from the machining area is increasingly evident with the machining time, during which the machined dimple is deeper and deeper, and, correspondingly, the occurrence of the re-contact of the reflected electrolyte with the nozzle wall becomes increasingly significant. However, the hydraulic jump and the electrolyte film thickness on the workpiece surface gradually reduce, and they almost disappear when the machined cavity is drilled to be a certain depth. This change trend is almost the same and occurs regardless of the electrolyte jet orientation. Furthermore, gravity does not showcase obvious asymmetry in the reflection of the impinged electrolyte in the horizontal orientation, due to the high velocity of electrolyte jet. In the initial phase of the electrolyte jet machining, re-contact hardly occurs, but the hydraulic jump is serious, so many electrolytes are accumulated on the workpiece surface with a very thick electrolyte film in all three examined jet orientations. However, in the deep-dimple phase, the flow behaviors and flow field characteristics differ markedly with the jet orientation. The horizontal orientation has a bigger reflection angle (the angle between the reflected electrolyte and the workpiece surface) than the vertically upstream orientation and the vertically downstream orientation—that is, where θ_Hm-D_ (the reflection angle of horizontal orientation in the deep-dimple phase) > θ_Vum-D_ (the reflection angle of the vertically upstream orientation in the deep-dimple phase) ≈ θ_Vdm-D_ (the reflection angle of the vertically downstream orientation in the deep-dimple phase).

**Figure 4:**
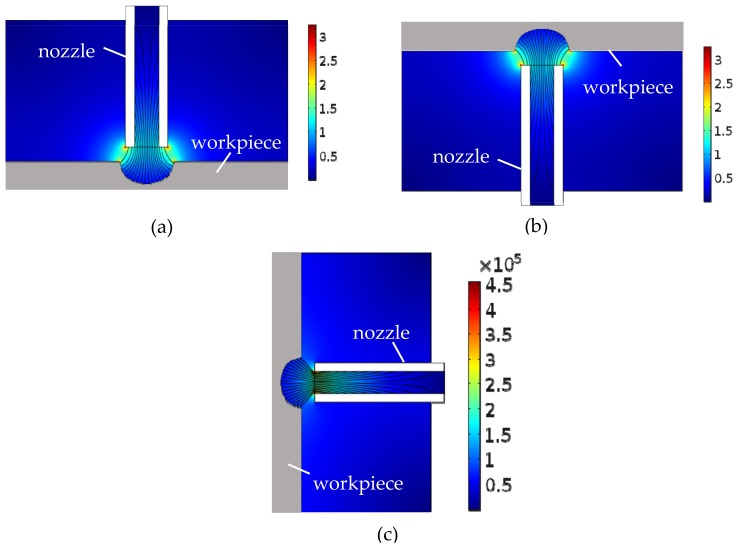
Simulated results of the current density distribution during Jet-ECM in the deep-dimple phase. (**a**) Vertically downstream orientation; (**b**) vertically upstream orientation; (**c**) horizontal orientation.

Figure 4 shows the simulated results of the current density distribution during Jet-ECM with three orientations in the deep-dimple phase. The current density distribution in the vertically downstream orientation and vertically upstream orientation are similar in the working gap, which is maximum at the outer wall of the nozzle and the edge of the micro-dimple, while the current density in the horizontal orientation is centralized at the inner wall of the nozzle, and the current is more concentrated. Compared to other jet orientations, the current density distribution near the tip of the nozzle decreases significantly in the horizontal orientation.

### 2.2. Experimental Observation of Flow Field Characteristics of Electrolyte Jet with Different Orientation

**Figure 5:**
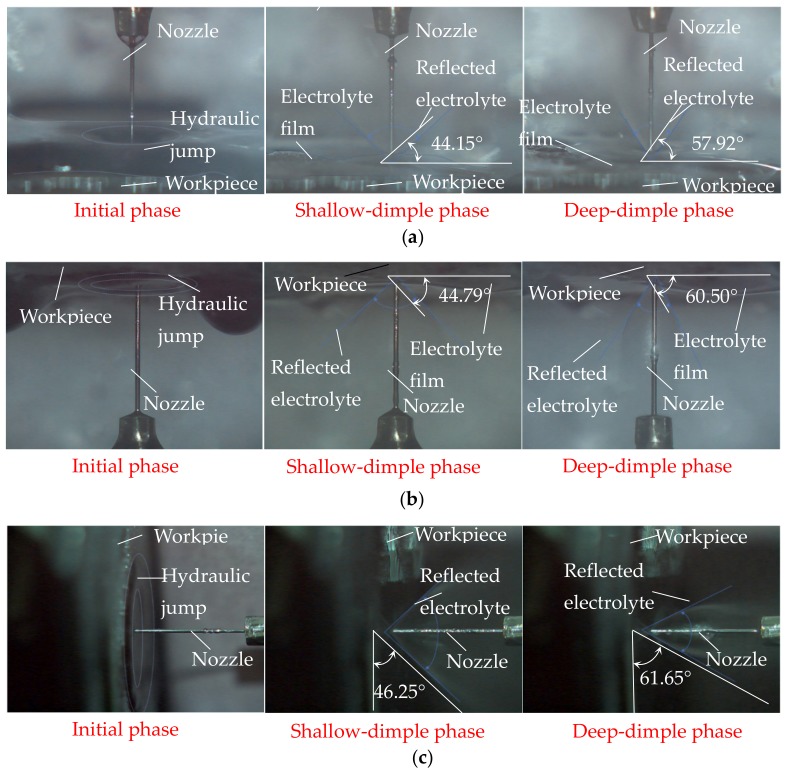
Observational results of the flow field during Jet-ECM in different phases when the electrical voltage was 15 V, the nozzle’s inner diameter was 170 μm, and the working gap was 100 μm. (**a**) Vertically downstream orientation; (**b**) vertically upstream orientation; (**c**) horizontal orientation.

To further clarify the effect of the jet orientation on the electrolyte flow behaviors during Jet-ECM, experimental observations (when the electrical voltage was 15 V, the nozzle’s inner diameter was 170 μm, and the working gap was 100 μm) were carried out, as shown in Figure 5. It was found, experimentally, that the hydraulic jump and the reflection of the electrolyte are both highly dependent on the machining phase with a given orientation and the orientation in the same machining phase. In the initial machining phase (when the machining time is 0 s), the hydraulic jump is most serious, with the thickest electrolyte film in the vertically downstream orientation, and it becomes much weaker in the other two orientations, so the electrolyte film is reduced. In the shallow-dimple phase (when the machining time is 7 s), the electrolyte reflection appears in all jet orientations, but its angle changes with the jet orientation, which is the smallest in the vertically downstream orientation (about 44.15°) and the biggest in the horizontal orientation (about 46.25°). Meanwhile, in this machining phase, the hydraulic jump becomes weaker for all jet orientations, and it can hardly be observed in the horizontal orientation, so the electrolyte film is very thin. In the deep-dimple phase (when the machining time is 15 s), which is considered to be a stable machining phase that mostly determines the machining characteristics of the Jet-ECM, the electrolyte reflection becomes more significant, and, thereby its angle is also bigger. Comparatively, the electrolyte reflection is most significant, with the biggest reflection angle being about 61.65°, in the horizontal orientation, and it is the weakest in the vertically downstream orientation, with the smallest reflection angle being about 57.9°. Meanwhile, in this a machining phase, the electrolyte film still exists in all the vertical jet orientations, but it disappears in the horizontal jet orientation, and, therefore, the left electrolyte is not observed on the workpiece surface.

Generally, the simulated results (shown in Figure 3) agree with the experimental findings, although the hydraulic jump and the electrolyte film thickness observed are not very consistent with those simulated. These differences could be from the assumptions set to the simulations.

The findings from both simulations and experimentations indicate that horizontal placement of the jet is able to provide the Jet-ECM process an improved mass transfer environment with almost no presence of electrolyte film on the workpiece surface surrounding the machining area, and the bigger reflection angle indirectly proves that the microcavities being machined with a horizontal electrolytic jet have a less tapered wall, and the current density is also more concentrated, i.e., it shows a better machining accuracy. The disappearance of the electrolyte film in the horizontal jet machining mode is caused by the promotion of a gravitational effect on the efficient removal of the electrolyte along the vertical surface.

## 3. Experimental

**Figure 6:**
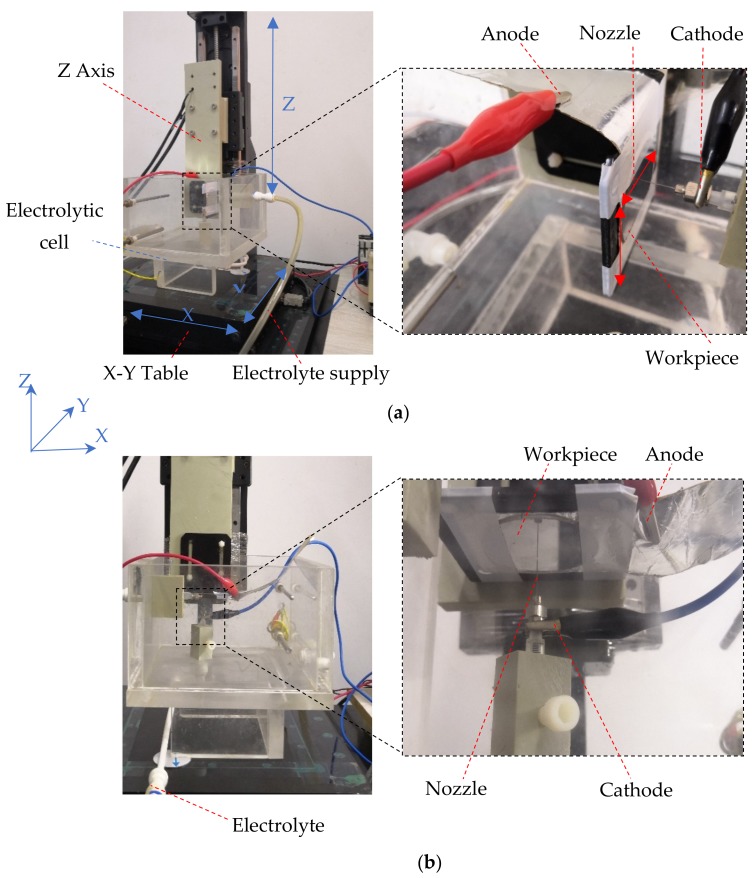
Jet electrochemical machining with different orientations. (**a**) Horizontal orientation; (**b**) vertically upstream orientation.

Experiments in this study were conducted in a specially designed apparatus, which is depicted in detail in [23]. To investigate the effects of the jet orientation on the Jet-ECM process, three jet orientations were designed, i.e., on in the vertically downstream orientation, on in the vertically upstream orientation, and one in the horizontal orientation. Correspondingly, three Jet-ECM machining modes were named here: the vertically downstream machining mode, the vertically upstream machining mode, and the horizontal machining mode. In the vertically downstream and vertically upstream machining modes, the electrolyte jet was impinged vertically downstream and upstream against the workpiece surface, respectively, and the surface to be machined was perpendicular to the impinging jet. In the horizontal machining mode, the electrolyte was impinged horizontally against the workpiece surface, and the surface to be machined was also perpendicular to the impinging jet. The above three machining modes are illustrated schematically in Figure 1. During machining, the workpiece was installed on an XY table, and the nozzle was mounted on a Z table for the vertically downstream mode or on specially designed fixtures for the vertically upstream mode and horizontal mode, as illustrated in Figure 6. The working gap between the workpiece and the nozzle tip could be adjusted controllably.

To fully assess the machining effects of Jet-ECM with different jet orientations, both static machining and translational machining were studied. In the static machining, the jet and the workpiece were kept static, and in the translational machining, the jet was translated over the workpiece. In this study, the electrolyte was 20 wt.% sodium nitrate (NaNO_3_) aqueous solution, because this kind of electrolyte has been proven to have a high machining accuracy. The output pressure of the electrolyte was 1 Mpa, and it should be greater than 0.5 Mpa to counteract the gravity force in the vertical upstream and horizontal orientations. The workpiece was stainless steel SUS304 with a mirror-like surface. The inner and outer diameters of the nozzle (SUS 304) from which the electrolyte jet was ejecting were 170 μm and 310 μm, respectively. The working gap was mostly kept at 100 μm in the static machining. Different micro-scale features were fabricated in this study. For micro-dimples, the applied voltage was in the range of from 6 V to 28 V, with an increment of 2 V, and the machining time for each micro-dimple was 15 s. Researchers chose larger voltages in related studies [24,25], but we selected a smaller electric voltage because the inner diameter of the nozzle and working gap was small. For micro-perforations, the applied voltage was 10 V, and the machining time for each perforation was also 15 s. For the micro-grooves fabricated with the translating machining, the applied voltage ranged from 10 V to 30 V, with an increment of 4 V, and the translating speed was 200 μm/s. Table 4 lists the main machining conditions and parameters.

A digital microscope (VHX-2000, Keyence, Osaka, Japan) and a scanning electron microscope (Merlin Compact, Carl Zeiss NTS GmbH, Oberkochen, German) were used to characterize the machined micro-structures. An interferometric surface profilometer (Talysurf CCI6000, Taylor, Slough, UK) was employed to measure the surface roughness of the machined surfaces.

## 4. Static Jet Electrochemical Machining Microstructures

### 4.1. Machining Micro-Dimples

#### 4.1.1. Surface Morphologies and Surface Roughness

**Figure 7:**
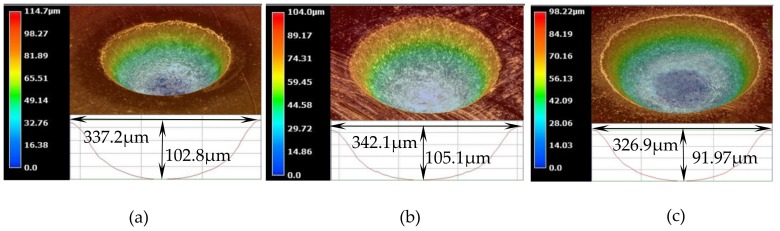
3D topographies of micro-dimples machined with different modes produced at 10 V. (**a**) Vertically downstream mode; (**b**) vertically upstream mode; (**c**) horizontal mode.

**Figure 8:**
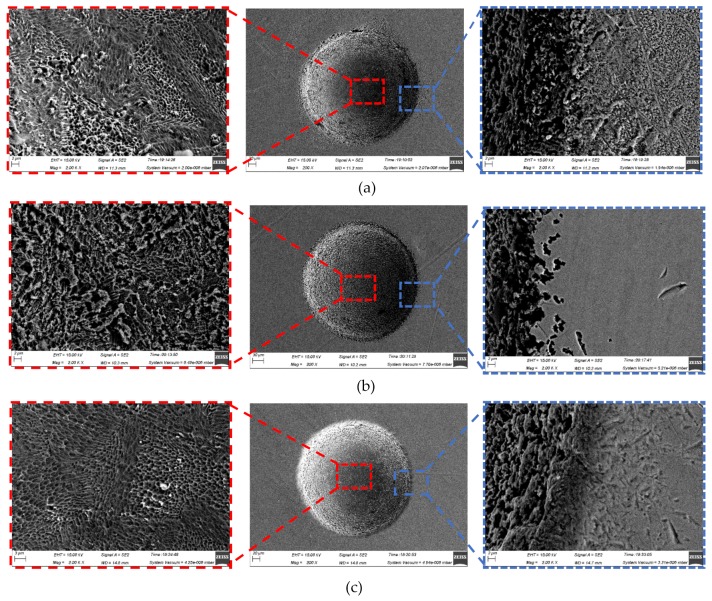
Scanning electron microscope (SEM) images of the micro-dimples machined with different modes when the electrical voltage was 10 V, the nozzle’s inner diameter was 170 μm, and the working gap was 100 μm. (**a**) Vertically downstream mode; (**b**) vertically upstream mode; (**c**) horizontal mode.

**Figure 9:**
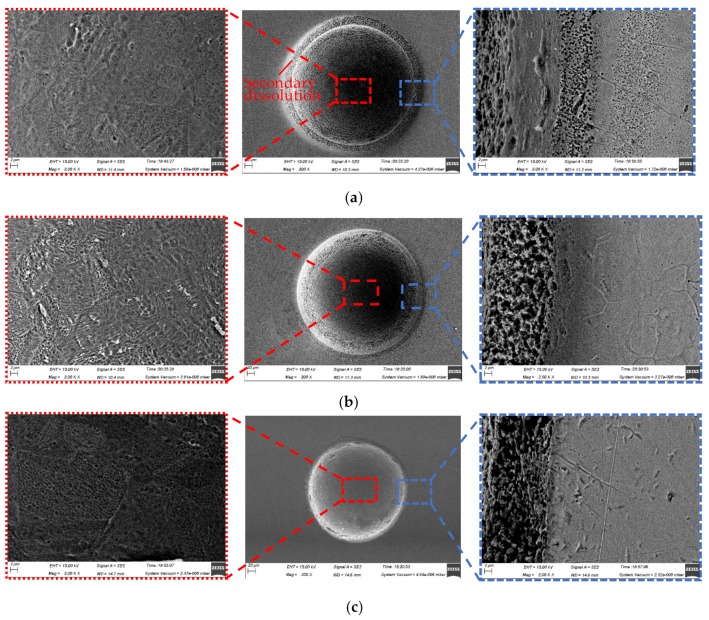
SEM images of the micro-dimples machined with different modes when the electrical voltage was 20 V, the nozzle’s inner diameter was 170 μm, and the working gap was 100 μm. (**a**) Vertically downstream mode; (**b**) vertically upstream mode; (**c**) horizontal mode.

**Figure 10:**
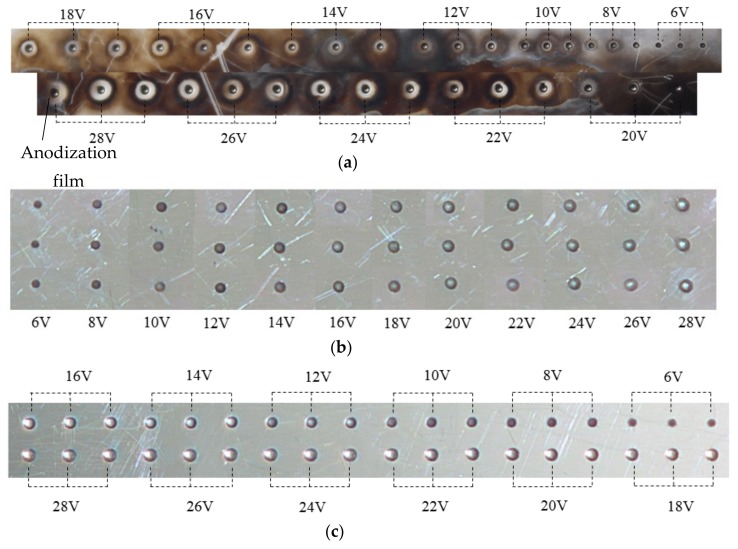
Photos of micro-dimples machined with different modes when the nozzle’s inner diameter was 170 μm and working gap was 100 μm. (**a**) Vertically downstream mode; (**b**) vertically upstream mode; (**c**) horizontal mode.

Although the three-dimensional geometric shape of the machined micro-dimples appeared to be highly identical regardless of the jet orientation (see Figure 7), the surface morphologies within the micro-dimples and the non-machining zone adjacent to the machined micro-dimples were considerably varied depending on the jet orientation during machining at 10 V (shown in Figure 8) and 20 V (shown in Figure 9). It was found that the micro-dimples formed with the horizontal jet orientation featured the smoothest surfaces, while the ones formed with the vertically upstream jet orientation showed the coarsest surfaces. These changes in the surface characteristics of the micro-dimples according to jet orientation can be further verified from the variations of the surface roughness of the machined micro-dimples produced at 10 V. The surface roughness, Ra, of the micro-dimples was 0.047 μm when the horizontal machining mode was used, but it increased to be 0.086 μm and 0.127 μm, respectively, when the vertically downstream machining mode and the vertically upstream machining mode were employed. These findings indicate that the use of the horizontal jet orientation improves the surface quality of the machined micro-dimples. The major reason for these differences could be the change in the machining mode of the Jet-ECM. As described above, in the horizontal machining mode where the electrolyte jet is orientated horizontally and the machined surface of the workpiece is orientated vertically, the hydraulic jump is almost absent, and, thus, no observable electrolyte film exists on the workpiece surface, which indicates that stray-current corrosion caused by an accumulation of the electrolytes and the electrolytic products is markedly reduced. A more concentrated current density distribution in the horizontal jet orientation makes the surface smoother. In contrast, in the vertically downwards machining, the hydraulic jump phenomenon is most significant, and the electrolyte film on the workpiece surface is thickest, meaning that the removal of the electrolytic products is seriously blocked, while in the vertically upstream machining mode, the hydraulic jump phenomenon occurs intermediately. The coarsest surface in the vertically upstream machining is related to the decentralized current density distribution and the difficult discharge of the machining products. The variation in the degree of stray-current corrosion that occurred at the non-machined surface with the jet orientation can be further demonstrated by the results illustrated in Figure 10, in which the anodization films (black film) formed on the areas adjacent to the machined micro-dimples are shown.

#### 4.1.2. Geometric Dimensional Accuracy

In order to analyze the effect of the jet orientation on the geometric dimensional accuracy of the micro-dimples, the diameter, depth (maximum), aspect ratio (depth/diameter), and the etch factor (EF) of the machined micro-dimples fabricated with different machining modes were calculated. Here, EF is introduced to further characterize the machining localization, which is frequently used in through-mask electrochemical machining [26]. EF is defined for the electrolytic jet machining processes as follows:

EF = 2h/(d − d_0_) (10)

where h is the maximum depth of the machined microstructures, d_0_ is the inner diameter of the nozzle, and d is the diameter of the machined micro-dimple. A bigger EF value means a better machining localization.

**Figure 11:**
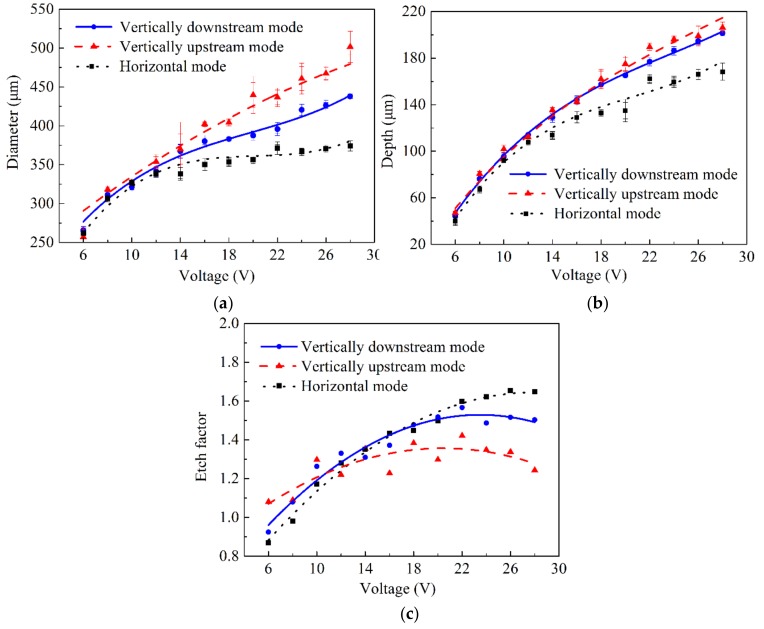
Change of the geometric dimensional sizes (diameter, depth, etch factor) of the machined micro-dimples with the applied voltage when the nozzle’s inner diameter was 170 μm and working gap was 100 μm. (**a**) Diameter; (**b**) depth; (**c**) etch factor.

Figure 11 shows the change of the geometric dimensional features of the machined micro-dimples with the applied voltage. In general, the horizontal placement of the jet enabled the Jet-ECM to achieve the smallest, and simultaneously the shallowest, micro-dimples at any given applied voltage, while the other two placements of the jet yielded different machining effects depending on the applied voltage. At low voltages of below 10 V, the geometric dimensions (depth and diameter) of the micro-dimples produced with the three machining modes showed few differences, but the etch factor changed with the jet orientation and was the largest when the jet was orientated vertically upstream. When the voltage was increased, the geometric dimensional features and EF values of the machined micro-dimples differentiated greatly depending on the jet orientation. With the vertically upstream mode, the micro-dimples were largest and deepest; with the horizontal machining mode, they were the smallest and shallowest, and with the vertically downstream machining mode, the diameter and depth of the micro-dimples showed intermediate values. Correspondingly, the EF value of the micro-dimples obtained with the vertically downstream machining mode was smallest and the one obtained with the horizontal machining mode was largest. Consequently, we found that the horizontal Jet-ECM possesses the best machining localization and has the smallest machining speed, whereas the vertically upstream Jet-ECM has the worst machining localization, and the vertical jet-ECMs have almost the same machining speed. These differences are primarily a result of the variations in the stray current and mass transfer conditions caused by the change of jet orientation.

### 4.2. Machining Micro-Holes

Since the mechanisms and processes of the hole-drilling before the holes are anodically penetrated almost the same as those of the dimple fabrication, in this study, we focused on investigating the effect of the jet orientation on the machining characteristics at the end phase of the Jet-ECM drilling micro-holes.

**Figure 12:**
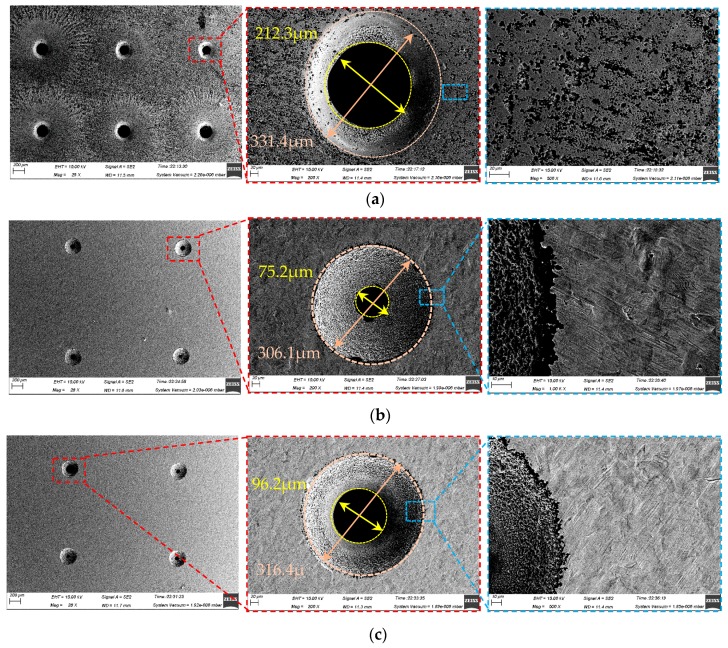
SEM images of the micro-holes machined with different modes when the electrical voltage was 10 V, the nozzle’s inner diameter was 170 μm, and the working gap was 100 μm. (**a**) Vertically downstream mode; (**b**) vertically upstream mode; (**c**) horizontal mode.

**Figure 13:**
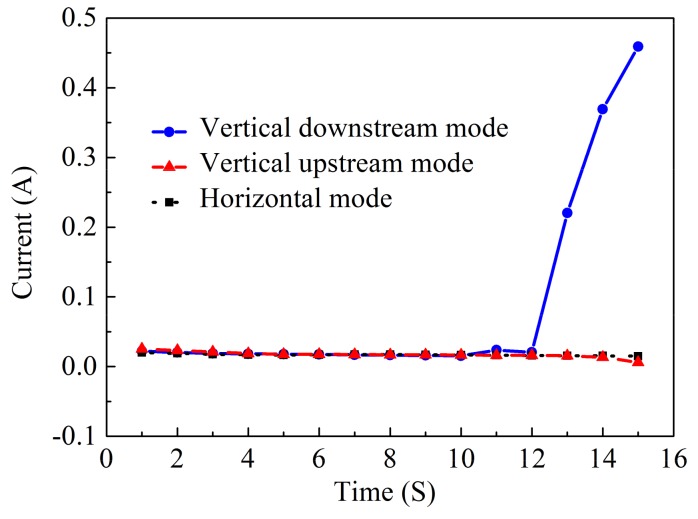
Machining current of the micro-holes machined with different modes when the electrical voltage was 10 V, the nozzle’s inner diameter was 170 μm, and the working gap was 100 μm.

Figure 12 illustrates the scanning electron microscope (SEM) images of the micro-holes formed with three different machining modes using the same process parameters. It was found by comparative analysis that the holes fabricated by the vertically downstream electrolytic jet had the biggest over-cut and outlet openings, as well as the roughest hole-wall surfaces, while the ones fabricated by the vertically upstreaming electrolytic jet showed the smallest over-cut and outlet openings, and a relatively good hole-wall surface. However, the ones fabricated by the horizontal electrolytic jet exhibited an intermediately big overcut and outlet opening but had the smoothest hole-wall surface. These variations may mainly be caused by a combination of the mass transfer conditions and interelectrode current distribution during the hole-penetration phase. As shown in Figure 13, the change in the interelectrode current with the drilling time depends considerably on the jet orientation. With the vertically downstream machining mode, the interelectrode current increased dramatically during the penetration-through period (in contrast with the other two modes) and remained almost unchanged during the entire machining time. Steadiness in the interelectrode current indicated a good stability in the anodic dissolution during the Jet-ECM and thus showed favorable machining effects, as shown in Figure 12b,c.

## 5. Fabrication of Microgrooves Using Translating Machining Method

**Figure 14:**
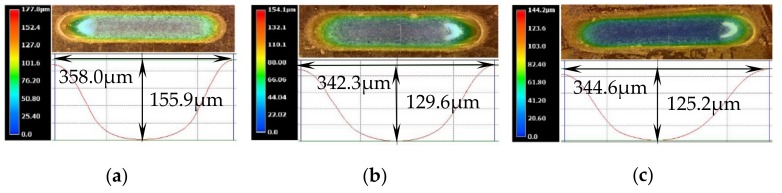
3D topographies of the micro-grooves machined with the translating mode produced when the electrical voltage was 30 V, the translating speed was 200 μm/s, the nozzle inner diameter was 170 μm, and working gap was 100 μm. (**a**) Vertically downstream mode; (**b**) translating vertically in horizontal mode; (**c**) translating horizontally in horizontal mode.

**Figure 15:**
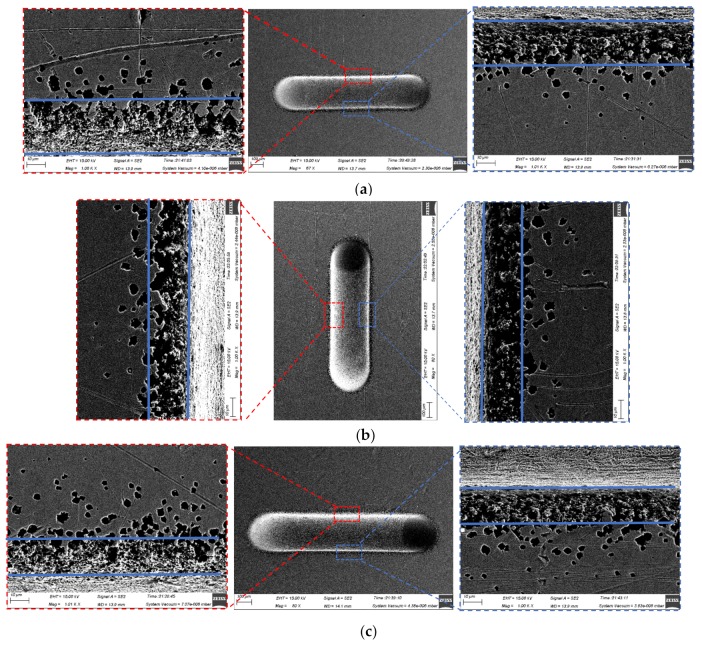
SEM images of micro-grooves machined with the translating machining mode when the electrical voltage was 20 V, the translating speed was 200 μm/s, the nozzle’s inner diameter was 170 μm, and the working gap was 100 μm. (**a**) Vertically downstream mode; (**b**) translating vertically in the horizontal mode; (**c**) translating horizontally in the horizontal mode.

**Figure 16:**
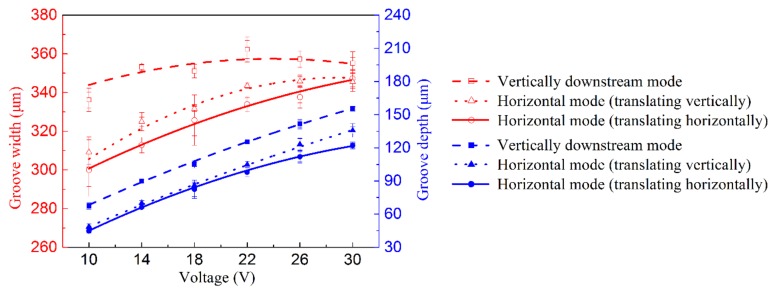
Change of geometric dimensional features (width, depth) of the machined micro-grooves when the translating speed was 200 μm/s, the nozzle’s inner diameter was 170 μm, and the working gap was 100 μm.

The topographies of the microgrooves machined with the translating machining method at 30 V are shown in Figure 14. The machined micro-grooves were highly similar in the three-dimensional geometric profiles regardless of either the translating direction (translating to and from vertically or horizontally) or the jet orientation (horizontal or vertically downstream), but, in effect, there were small differences in their geometric dimensions. With a jet orientated horizontally, the generated microgrooves were narrower and shallower (see Figure 14b,c), showing a higher machining localization but a lower machining speed. The reasons for these discrepancies could be the same as those explained previously for the static machining mode: much less stray-current corrosion occurred, and the mass transfer was more efficient when the jet was orientated horizontally. The SEM images of the microgrooves, shown in Figure 15, further prove the effects of the jet orientation and translation direction on the dissolution behaviors of the Jet-ECM. The microgroove formed with the translated jet was orientated horizontally showed slightly corroded morphologies on its entrance regions (see Figure 15b,c) caused by a stray current. However, the one formed with the vertically downstream jet showed seriously corroded entrance regions (see Figure 15a). Further, the data from Figure 16, which quantitatively shows the effect of the jet orientation and translation direction of the Jet-ECM on the machining accuracy of the microgrooves, prove the conclusion that a translating machining method with a horizontally-orientated jet possesses a higher machining localization and a smaller machining speed, irrespective of the jet-translation direction.

## 6. Conclusions

To better understand and utilize the jet electrochemical machining processes, this paper systemically investigated the machining characteristics with different jet orientations, including a vertically downstream orientation, vertically upstream orientation, and horizontal orientation. The Jet-ECM with the latter two jet-orientations has rarely been evaluated. Some simulations and experiments were carried out to analyze the flow field and current density distribution characteristics and, further, to elucidate the differences in dissolution behaviors, material removal rates, machining accuracy, and surface characteristics of the formed features with the jet orientation of the Jet-ECM. In the following, some conclusions from this study are listed.

(1) The machining accuracy and surface morphologies of the formed micro-sized features, and material removal rate (MMR) are highly dependent on the jet orientation of the Jet-ECM.
(2) The horizontal jet orientation is of great benefit to achieving accurate micro-sized features with excellent surface quality with either a static jet or a scanning jet for Jet-ECM, while the Jet-ECM processes with vertical jet orientations, including a vertically downstream orientation and upstream jet orientation, are not able to produce favorable microfeatures.
(3) The Jet-ECM with a horizontal jet orientation has a smaller MMR than the ones with vertical jet orientations, which have almost the same MMR.
(4) Enhancement of the machining localization and reduction of the MMR for horizontal jet electrochemical machining result largely from the improvement of the mass-transfer field. With the horizontal jet orientation, the reflection angle of the electrolyte is very big. Almost no hydraulic jump is observed, and, thus, the electrolyte film is extremely thin for the positive effect of the gravity, which indicates that there is little blockage of the mass transfer. Furthermore, the current density is more concentrated in the horizontal jet orientation.

## Figures and Tables

**Table 1:** Domain and boundary conditions for the simulations of fluid dynamics.

**Domain Conditions**	**Domain**	**Property**
Fluid property	I	Electrolyte
Nozzle materials	II	SUS304
Fluid property	III	Air
Initial values electrolyte	I	u_ini_ = 0, P_ini_ = 0
-	III	u_ini_ = 0, P_ini_ = 0
Gravity	I, III	g_x_ = 0, g_y_ = −g
**Boundary Conditions**	**Boundary**	**Property**
Initial interface	5	-
Inlet electrolyte	1	Laminar inflow, Φ = 1, u = 15 m/s
Outlet	2,3,6,7	P = 0 Pa

**Table 2:** Material definitions for the simulations of electrodynamics.

Domain	Material	Parameter
I	Electrolyte	σ_E_ = 16 S/m
III	Air	σ_A_ = 1 × 10^−5^ S/m

**Table 3:** Boundary conditions for simulations of electrodynamics.

Boundary	Boundary Condition	Parameter
4	Electric potential	U_A_ = 20 V
Nozzle wall	Electric potential	U_C_ = 0 V

**Table 4:** Experimental conditions.

Parameters	Value or Variable
Electrolyte	NaNO_3_ aqueous solution
Concentration (wt.%)	20%
Electrolyte temperature (℃)	25
Output pressure (MPa)	1
Nozzle inner diameter (μm)	170
Working gap (μm)	100
Workpiece material	Stainless steel SUS304
Nozzle material	Stainless steel SUS304
Electrical voltage (V)	6–30
Translating speed (μm/s)	200
